# Phytochemicals against TNF*α*-Mediated Neuroinflammatory Diseases

**DOI:** 10.3390/ijms21030764

**Published:** 2020-01-24

**Authors:** Lalita Subedi, Si Eun Lee, Syeda Madiha, Bhakta Prasad Gaire, Mirim Jin, Silvia Yumnam, Sun Yeou Kim

**Affiliations:** 1 College of Pharmacy, Gachon University, #191, Hambakmoero, Yeonsu-gu, Incheon 21936, Korea; subedilali@gmail.com (L.S.); dltldms90@nate.com (S.E.L.); samarpanbp@gmail.com (B.P.G.); 2 Neurochemistry and Biochemical Neuropharmacology Research Unit, Department of Biochemistry, University of Karachi, Karachi-75270, Pakistan; syedamadiha2010@live.com; 3 College of Medicine and Department of Health Science and Technology, GAIHST, Gachon University #155, Gaebeol-ro, Yeonsu-gu, Incheon 21999, Korea; mirimj@gachon.ac.kr

**Keywords:** TNF-α, TNFR1, TNFR2, neuroinflammation, neurodegeneration, phytochemicals

## Abstract

Tumor necrosis factor-alpha (TNF-*α*) is a well-known pro-inflammatory cytokine responsible for the modulation of the immune system. TNF-*α* plays a critical role in almost every type of inflammatory disorder, including central nervous system (CNS) diseases. Although TNF-α is a well-studied component of inflammatory responses, its functioning in diverse cell types is still unclear. TNF-*α* functions through its two main receptors: tumor necrosis factor receptor 1 and 2 (TNFR1, TNFR2), also known as p55 and p75, respectively. Normally, the functions of soluble TNF-α-induced TNFR1 activation are reported to be pro-inflammatory and apoptotic. While TNF-*α* mediated TNFR2 activation has a dual role. Several synthetic drugs used as inhibitors of TNF-*α* for diverse inflammatory diseases possess serious adverse effects, which make patients and researchers turn their focus toward natural medicines, phytochemicals in particular. Phytochemicals targeting TNF-*α* can significantly improve disease conditions involving TNF-*α* with fewer side effects. Here, we reviewed known TNF-*α* inhibitors, as well as lately studied phytochemicals, with a role in inhibiting TNF-*α* itself, and TNF-*α*-mediated signaling in inflammatory diseases focusing mainly on CNS disorders.

## 1. Introduction

Cytokines are involved in autocrine, paracrine, and endocrine functions, and are either pro-inflammatory or anti-inflammatory in nature. Pro-inflammatory cytokines are involved in both the induction and progression of inflammatory reactions or pathologies, including inflammation, pain, and cancer [1]. Among such inflammatory cytokines, tumor necrosis factor-alpha (TNF-*α*) is a well-known pro-inflammatory cytokine responsible for the modulation of the immune system. TNF-α is synthesized as a type II transmembrane protein made up of 233 amino acids, which then undergo proteolytic cleavage through TNF-*α* converting enzyme (TACE) to form active TNF-*α* [2]. Various human cells are capable of producing TNF-*α*, especially macrophages and monocytes. Enhanced TNF-*α* production can alleviate cellular signaling that can cause cells to undergo necrosis or apoptosis. Despite roles in defense mechanism against inflammatory conditions, TNF-*α* is well characterized as a pathogenic mediator in diverse inflammatory diseases, including Alzheimer’s disease (AD), Parkinson’s disease (PD), stroke, psoriasis, arthritis, septic shock, and pulmonary disorders [3,4,5]. 

The biological functions of TNF-*α* are mediated through its two main receptors: tumor necrosis factor receptor 1 (TNFR1) (p55) and tumor necrosis factor receptor 2 (TNFR2) (p75) [6]. Activation of TNFR1 is known to initiate inflammatory, apoptotic, and degenerative cascades, whereas TNF-*α* signaling through TNFR2 is anti-inflammatory and cytoprotective, resulting in the induction of proliferation, differentiation, angiogenesis, and tissue repair [7,8]. Soluble TNF-*α* and transmembrane TNF-α are the two main forms of TNF-*α*, and each has a distinct role and signaling pattern. Soluble TNF-α is usually considered to have inflammatory effects through binding to its TNFR1 receptor, whereas transmembrane TNF-*α* preferentially binds to TNFR2 receptors and exerts anti-inflammatory effects. Binding of TNF-*α* to TNFR1 can initiate cell apoptosis through activation of mitogen activated protein kinase (MAPK), caspases, and transcription through NF-κB (Nuclear factor kappa-light-chain-enhancer of activated B cells) signaling, which is responsible for cell death and pro-inflammatory conditions [9]. These receptors, as well as TNF-α, are well expressed/present in brain tissues [10]. TNFR1 mediated signaling induces the activation of PI3K (Phosphoinositide 3-kinase) signaling, which further activates caspase 8/3 and BH3 interacting-domain death agonist (BID). This is followed by the induction of oxidative stress, necrosis, and apoptosis [11,12], which are the main causes of neurodegeneration. TNFR2 activation by TNF-*α* is reported to increase cell survival as well as re-myelination of degenerated neurons in multiple sclerosis (MS) lesions, supporting the hypothesis that TNFR2 plays an opposite and beneficial role to that of TNFR1 in animal and human physiology [13]. When TNF-*α* binds to TNFR2, it can activate CXC motif chemokine 12 (CXCL12)/CXC chemokine receptor type 4 (CXCR4), responsible for the proliferation, differentiation, and re-myelination of the demyelinated neurons in MS lesions [14]. TNFR2-mediated activation of PI3K/AKT (Protein kinase B) and vascular endothelial growth factor receptor 2 (VEGFR2) is responsible for angiogenesis [15], and NADPH oxidase 4 (Nox4)/reactive oxygen species (ROS)-mediated heme oxygenase 2 (HO-2)/CO production, which is controlled by TNFR2, can induce cell survival during various injuries or insults in organs [16]. TNF-*α*/TNFR2 is also able to activate PI3K/VEGFR2 signaling, and this pathway causes angiogenesis, as Nox4/ROS-mediated HO-2/CO activation is responsible for cell survival and can inhibit neurodegeneration. Signaling via this pathway can also produce anti-inflammatory effects [15,17].

Neuroinflammation is inflammation of the neurons where cells of the central nervous system (CNS), such as neurons, macroglia, and microglia are involved. Microglia are the resident macrophage of the CNS and determine the fate of other neural cells. Upon encountering an endogenous or exogenous stimuli microglia are activated and initiates neuroinflammation by secreting various pro-inflammatory cytokines, such as TNF-*α*, interleukin-1*β* (IL-1*β*), IL-6, IL-18, reactive oxygen, and nitrogen species [18,19]. Neuroinflammation is an important feature of most of the neurodegenerative diseases, including Alzheimer’s disease (AD), Parkinson’s disease (PD), multiple sclerosis (MS), Huntington’s disease (HD), and amyotrophic lateral sclerosis (ALS). The pro-inflammatory cytokines, TNF-*α*, are an important component of neuroinflammation, and play both homeostatic and pathophysiological roles in CNS. In healthy CNS, TNF-α regulates many physiological processes, including sleep, learning and memory, synaptic plasticity, and astrocyte-induced synaptic strengthening. [20,21]. While in pathological conditions, microglia and astrocytes release large amounts of TNF-*α*, leading to the release of neuroinflammatory cascades that are associated with several neurodegenerative disorders. Miscontrol of inflammatory signaling has been involved in the pathogenesis of several neurological disorders, including AD and PD. Increase of TNF-*α*, a central mediator of neuroinflammation, occurs with the onset of early neurological diseases, which develop typical pathologies in age-related neurological diseases [22]. Additionally, TNF-*α* and TNFR1 in neurodegenerative disorders also contribute to amyloidogenesis [23]. Therefore, TNF-*α* is a promising candidate for future TNF-*α* -based neuroinflammation therapy. This review summarizes the role of TNF-α in neuroinflammation and discusses various phytochemicals that inhibit TNF-*α* and its neuroprotective mechanism against neurodegenerative diseases.

## 2. TNF-*α* Signaling in Neuroinflammation

**Figure 1:**
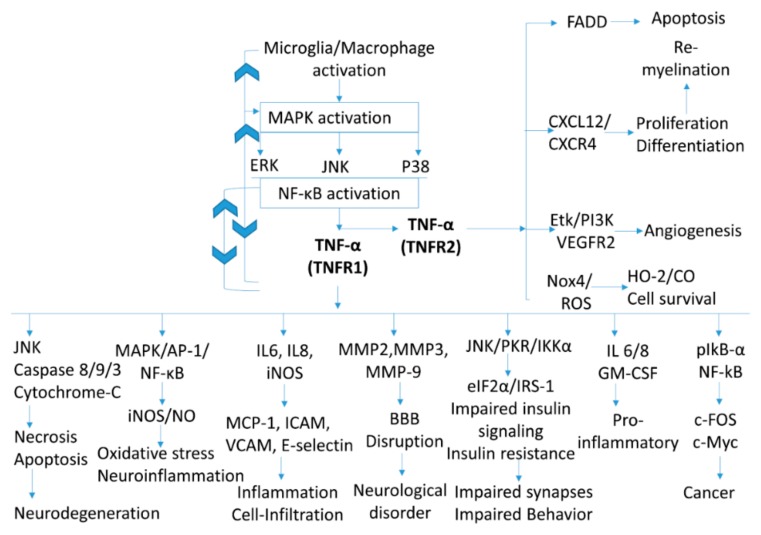
Receptor-mediated endogenous signaling pathways of tumor necrosis factor-alpha (TNF-*α)*.

In neuroinflammatory disorders, secretion of large amounts of TNF-*α* from microglia is mostly responsible for conditions such as neuroinflammation and excitotoxicity [24]. Astrocytes and neurons can also express TNF-*α* receptors and secrete TNF-*α*, which can trigger inflammatory cascades following neurological disorders. However, microglia cells are a major source of TNF-*α* in the brain compared to other cells [21]. Moreover, neuroinflammatory disease as well as neurodegenerative disorders are characterized by extensively elevated levels of pro-inflammatory cytokines, including TNF-*α*, IL-1*β*, IL-6, and IL-18. These overall cascades begin by TNF-*α* binding and are summarized in Figure 1. Moreover, increased levels of TNF-*α*, IL-1*β*, and nerve growth factor (NGF) were detected in the inflamed paws of mice. Administration of an anti-TNF-*α* antibody significantly reduced the levels of TNF-*α* and IL-1*β*, followed by a reduction in paw inflammation [25]. TNF-*α* binding with its cell surface receptor upregulate mitogen activated protein kinase (MAPK) signaling. MAPK signaling includes p38, extracellular-signal-regulated kinases (ERKs), and cJun NH2-terminal kinases (JNKs). MAPK signaling activation leads upregulation of the production of pro-inflammatory cytokines, such as IL6, IL-1*β*, and TNF-*α* as a secondary response. TNF-*α* increased in this way is responsible for the biological activity [26]. TNF-*α*-mediated stress stimuli induce activation of ERK, p38, and JNK, non-specifically. Stress-activated MAP kinase signaling, such as p38 and JNK are dramatically upregulated following TNF-*α* treatment pathways [27]. This mechanism of JNK and p38 MAPK pathway activation has been associated with sustained TNF*α* signaling during the cell death response [26]. On the other hand, activation of the JNK through any stress stimuli can actively participate in the macrophage activation towards the inflammatory M1 phenotype via increased TNF-α. Hence, JNK activation is believed to be involved in the secretion of the TNF-*α* and the neuroinflammatory cascades [28]. In addition, TNF-*α*-mediated cytokine production, followed by intracellular adhesion molecule 1 (ICAM-1), vascular cell adhesion protein 1(VCAM-1), E-selectin, and P-selectin expression, can cause cell infiltration and inflammation [29]. Increased levels of TNF-*α* in the blood activates matrix metalloproteinase 9 (MMP-9), which causes blood-brain barrier (BBB) disruptions and induces related neurological disorders [30].

Furthermore, induction of insulin resistance is an unwanted condition, both in terms of neurological and diabetic complications. TNF-*α* is capable of inducing such conditions via JNK/protein kinase C-related kinase (PKR)/IKK*α* (IkappaB kinase alpha) signaling-mediated disturbances in eukaryotic initiation factor 2-alpha (eIF2*α*)/insulin receptor substrate 1 (IRS-1) insulin signaling, and causing impaired synapses, as well as impaired behavioral control in the brain [31,32,33]. Activation of c-Fos and c-Myc through TNF-*α*-activated phospho (p)-IκB-*α*/NF-κB is responsible for carcinogenicity [34]. Previous studies revealed that TNF-*α* mediates signaling via various pathways such as JNK/IKKB (Inhibitor of nuclear factor kappa-B kinase subunit beta), which is responsible for AD pathology [35], NF-κB/activator protein 1 (AP-1) for PD, TACE/SOD1 (Superoxide dismutase 1) for ALS, and caspases for HD [36]. 

## 3. TNF-*α* in Neurodegenerative Disorders

TNF-*α*-mediated elevation in amyloid beta (A*β*) plaque burden and *β*-secretase 1 (BACE1) expression is responsible for abnormal A*β* processing. This abnormal processing induces synaptic loss followed by neuronal loss and neuronal cell death, ultimately causing dementia-characterizing AD lesions [37]. Additionally, TNF-*α*-mediated transcytosis allows TNF-*α* to permeate easily through the BBB, causing further BBB disruption and AD pathology [38]. Phase I and IIa clinical trials on TNF-α inhibitors have revealed their role in controlling cognitive and memory decline in AD patients. The supporting evidence clearly suggests the role of TNF-*α* in the pathology of AD, and inhibition of TNF-*α* production or expression cannot only obstruct the disease pathology, but also prevent further damage and severity [22].

PD is characterized by the loss of dopaminergic neurons, neuroinflammation, and toxicity-induced neuronal death. Over-activated glial cells, such as microglia and astrocytes, are responsible for the production of a number of inflammatory mediators or pro-inflammatory cytokines, including TNF-α, which can induce neuronal death and conditions, under which continuous degeneration or death of the dopaminergic neurons can occur [39]. TNF-*α*-mediated MAPK or NF-κB/activator protein 1 (AP-1) signaling target the induction of apoptosis through various apoptotic pathways. In MD, activation of JNK by increased TNF-*α* is responsible for apoptosis and myelin degeneration [40]. In addition, the activation of TNFR2 by TNF-*α* may play a role in increasing angiogenesis, proliferation, differentiation, and re-myelination of degenerative or demyelinated axons of the neurons through CXCL12/CXCR4 in the CNS, especially in astrocytes [14].

**Figure 2:**
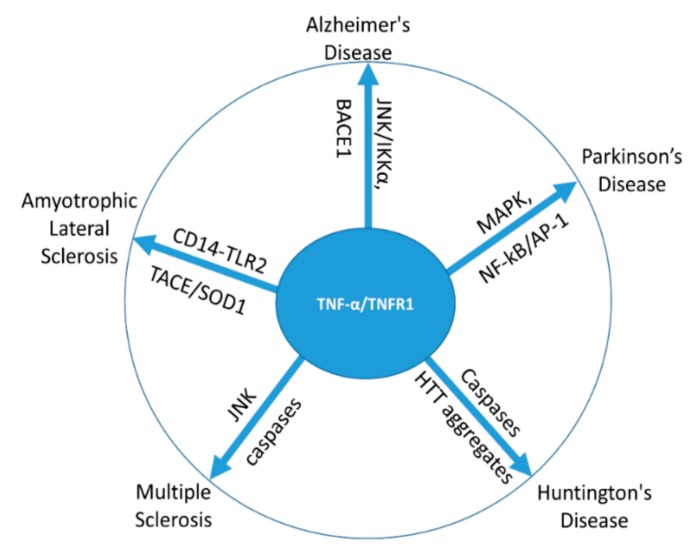
Involvement of TNF-*α* in various neurological disorders via action on different targets.

TNF-*α* is considered to be one of the key pathogenic factors that participate in the initiation and progression of the ALS pathogenesis. High levels of TNF-*α* and its soluble receptors TNFR1/TNFR2 were observed in the blood, cerebrospinal fluid, and nerve tissues of ALS patients and animal models [41]. Thalidomide and lenalidomide, which are TNF-*α* inhibitors, had been tested for ALS; however, they could not pass clinical trials [42]. Pathologically, mutation of SOD1 in ALS raises interest to find its interconnection with ALS as a first target [43]. It was revealed that extracellular SOD1 is not directly involved in the pathogenesis of ALS; however, it utilizes CD14 (Cluster of differentiation 14)-TLR2 pathway, which is triggered through the activated microglia-mediated release of TNF-*α* [44] that subsequently aggravate motor neuron degeneration, a characteristic feature in ALS. Further, activation of the ionotropic purinergic receptor P2X7 in SOD1^G93A^ microglia trigger the production of high levels of TNF*α*, which exert neurotoxic effects on the motor neuron [45], contributing to the pathogenesis of ALS. In addition, high levels of TNFRs are observed in the spinal cords of mutant SOD1 mice, which are associated with the activation of diverse signaling involving MKK3-6, MKK4, ASK1 (Apoptosis signaling-regulating kinase 1), and phosphorylated p38 MAPK (p-p38) in motor neurons and glial cells, which ultimately lead to the degeneration of motor neurons [41]. Altogether, these previous reports indicate that SOD/TNFRs/ASK1/p38 MAPK signaling have actively participated in spinal motor neuron degeneration associated with ALS. In addition, accumulation of SOD1-induced iron in the ventral motor neuron influenced the enzymatic activity of TACE, resulting in excessive TNF-*α* production [46]. TNF-*α* production-mediate glutamate excitotoxicity is also the prime cause for the motor neuron toxicity [41]. Collectively, TNF-*α* has a critical role in motor neuron degeneration, the major pathogenesis in ALS. The overall roles of TNF-*α* in various neurological disorders are summarized in Figure 2.

TNF-α also plays a dominant role during the inflammation in non-neuronal disorders. In case of diseases, such as rheumatoid arthritis (RA), Crohn’s disease (CD), psoriasis, retinitis, multiple myeloma, diabetes, obesity, human immunodeficiency virus (HIV-AIDS)-mediated inflammations, and cognitive impairment, anti-TNF agents are the first drug of choice to lower the inflammation [47,48]. This suggests that phytochemicals, having the potential to lower the secretion or inflammatory cascades of TNF-*α*, could be a great alternative for the treatment of immune mediated inflammatory conditions. Targeting TNF-*α* by natural lead compounds can be beneficial, not only for lowering neuroinflammation, but non-neuronal inflammation as well.

## 4. Commercially Available TNF-*α* Inhibitors and Their Side Effects

Many commercialized TNF-*α* inhibitors are available, such as adalimumab, apratastat, certolizumab, golimumab, infliximab, minocycline, thalidomide, GW333, BMS-561393, and etanercept (listed in Table 1). These drugs have roles in treating many disease conditions, including neurological disorders. However, the severe side effects of these inhibitors are the key reason for the withdrawal of such medicines. Such drugs can cause significant and even life-threatening adverse effects and are accordingly blacklisted. The three main negative effects of TNF-*α* inhibitors are as follows: (1) serious infections such as erysipelas, abscess, and candidiasis, (2) neoplasms, including squamous cell carcinoma and basal cell carcinoma, and (3) a very rare type of hepatosplenic T-cell lymphoma [49,50]. Apratastat is a well-known TACE and MMP inhibitor that is mostly prescribed for inflammatory joint disorders, but its approval has been terminated after clinical trials, because of its lack of efficacy [51]. Certolizumab shows potency against CNS infection and CD, where it inhibits TNF-α binding and hence inhibits TNF-*α*-mediated toxicity and inflammatory cascade activation. There are known side effects, these include moderate pain, abdominal disorders, injection site reaction, skin rashes, and urinary tract disorders, while serious infections, malignancies, and heart failure are severe side effects that have also been reported [52,53]. Similarly, golimumab and infliximab are also very important TNF-*α* inhibitors, which bind to soluble and transmembrane forms of TNF-*α* and block it from binding to its receptors [54,55]. These effects are useful for the treatment of conditions such as RA, psoriasis, psoriatic arthritis, ulcerative colitis, CD, and Wegener’s granulomatosis [54,55]. Besides such benefits, treatment of many disorders with compounds such as adalimumab, certolizumab, golimumab, and infliximab produces many side effects, including serious infections, such as a lower respiratory tract infection, skin infection, and tuberculosis [54], as shown in Table 1. Similarly, minocycline (a TNF-*α* synthesis inhibitor) and thalidomide (a TNF-*α* degradation inducer) also show side effects. Minocycline is normally prescribed for the treatment of ALS, MS, AD, stroke, traumatic brain injury (TBI), and spinal cord injury, while thalidomide is used to treat multiple myeloma, CD, human immunodeficiency virus (HIV), lupus, and leprosy. Their adverse effects are also well known in many treatment therapies. Minocycline mediates vestibular side effects, as well as leukopenia and weight loss, which are more specifically associated with females [56]. The adverse effects associated with thalidomide are more serious: thalidomide toxicity includes deep vein thrombosis, teratogenicity, constipation, pyrexia, fatigue, osteonecrosis of the jaw, pain, peripheral neuropathy (PN), and somnolence [57]. Recently, morphea was observed as a new side effect of TNF-*α* inhibitors [58]. In addition, the drug interactions of TNF-*α* inhibitors with methotrexate, anakinra, and abatacept [54,59] are another shortcoming of the known TNF-*α* inhibitors [60]. The immunogenicity, autoimmune disorders, and congestive heart failure caused by TNF-*α* inhibitors are also some negative effects associated with these drugs. Five main TNF blockers (etanercept, infliximab, adalimumab, golimumab, and certolizumab) are approved for clinical use, but the immune suppression and demyelination of the central and peripheral nervous system by these candidates, while using not only against neuronal disorders, but also against systemic inflammation, raise a big question for their safety profile and usability in the future, suggesting the importance of phytochemicals having potential, similar to that of TNF-*α* inhibitors/blockers with lesser side effects and better usability[61]. 

## 5. Phytochemicals Inhibiting TNF-*α* for Lowering the Neuroinflammatory and Neurodegenerative Disorders

TNF-*α*, being a key target that can modulate the pathology of multiple neurological disorders, has gathered enormous attention in the recent past. However, the serious side effects of commercial TNF-*α* inhibitors bring question to their efficiency, and there is increasing effort to screen TNF-*α* inhibitors/blockers from natural products, phytochemicals, and nutraceuticals. Novel TNF-*α* inhibitors/blockers may prove to be an alternative way of treating disorders in which TNF-*α* is involved as a key player. Many phytochemicals, such as curcumin, shogaol, paradol, and equol are known to have crucial roles in inhibiting TNF-*α* with lesser side effects [70,71,72,73]. Recent findings have suggested that phytochemicals, such as allyl isothiocyanate (AITC), quercetin, and kaempferol show potential to control neuronal disorders by inhibiting TNF-*α* production, as shown in Table 2. In addition, 6-shogaol, gingerol, and their derivatives from *Zingiber officinale*, bear great potential for limiting TNF-*α* activity, either by inhibiting binding or inducing degradation. Apigenin, naringenin, and myricetin also inhibit inflammatory cascades in diverse inflammatory disorders through significant inhibition of TNF-α expression. Moreover, we have identified many phytochemicals that show anti-inflammatory activity either via the inhibition of TNF-*α* binding and activity, or by direct inhibition. Most of these phytochemicals inhibit the production of TNF-*α* via inhibition of the NF-κB mediated transcription regulated by MAPK or PI3K signaling. Butein and hesperetin inhibit TNF-α secretion. Wogonin, morin, chrysin, eudesmin, mandolin and honokiol inhibit TNF-α through regulation of JAK/STAT3 (Janus kinase/signal transducer and activators of transcription) pathway or PI3K/Akt/MAPKs-NF-κB pathways, suggesting them to be possible candidates against AD pathology. Ginsenoside compounds from *Panax ginseng* also inhibit TNF-*α* significantly with subsequent inhibition of the inflammatory cascades, suggesting the phytochemicals as potent anti-inflammatory candidates. On a similar note, phytochemicals, such as nicotine, berberine, capsaicin, and kavalactone inhibit the TNF-*α* with concomitant amelioration of inflammatory and oxidative stress, resulting in anti-inflammatory effects, making them good candidates for inhibition of inflammation during AD and PD pathologies. Diallyl sulfide, present in *Allium sativum*, is also reported to have a strong anti-inflammatory effect via downregulating production of pro-inflammatory cytokines, such as TNF-*α* [74]. Similarly, curcumin, a major spice used in Asian countries, such as India, Nepal, and Pakistan, is a well-reported anti-inflammatory agent, having the potential to lower most pro-inflammatory mediators, including TNF-*α*. Many of the *in vitro* and *in vivo* studies have been performed regarding this potent compound; however, the solubility issues kept this in the shade [70,75,76]. Designing the better formulation could enhance the utilization of curcumin against several inflammatory conditions, including neuroinflammation and neurodegeneration, in which TNF-*α* could be the major pathogenic target. Overall, phytochemicals isolated from natural resources with the ability to inhibit TNF-*α* binding to TNFR1 and/or inhibit TNF-*α* activity could be vital candidates for the treatment of a number of neuroinflammatory disorders in humans. Table 2 summarizes the key phytochemicals that are reported to have a TNF-*α* inhibitory effect in diverse experimental models, indicating their potential as plausible anti-TNF-*α* therapy.

TNF-*α* mediated inflammatory cascades can be lowered by four main mechanisms, such as (1) by inhibiting the binding of TNF-*α* to TNFR1, (2) by lowering the activation of TACE that lowered the TNF-*α* production, (3) lowering the activation signaling that are responsible for TNF-*α* production, and (4) TNF-*α* degradation. Above mentioned medicinal plants, and their active chemicals target in one/more of these pathways and hence lowers the TNF-*α* and its related inflammatory pathways. Though the exact mechanism of those phytochemicals to inhibit the TNF-α is not known yet, most of them significantly lowered the TNF-*α* secretion either *in vitro* in the activated microglia/macrophage cells or in-vivo in the mice model of inflammation, respectively. These phytochemicals are in need of more research to explore their exact role to lower the TNF-*α* secretion and its inflammatory activity in cells and animal models. Only then could we reach closer to the TNF-*α* inhibiting drug discovery from natural products that could be effective, from the experimental table to the patient’s bed, in the near future.

Natural products are still considered to be a major source of lead compounds with desired pharmacological effects, those could be possible candidates for the drug discovery against several biological ailments covering inflammation to cancer [127]. However, research on natural products are mostly limited in the *in vitro* and *in vivo* studies, and very few have reached clinical trials, and even failed there [128]. These difficulties of drug discovery from natural resources have been because of various limitations of natural products, including complex molecular structure, solubility issues, the selectivity of the compounds to certain targets, and the specificity of the compounds towards experimental species [128,129]. The solubility issue of the phytochemicals rendering their activities can be achieved by designing better formulation through nanoparticle formulation, drug micelle formation, prodrug formulation, designing semisynthetic derivatives with the addition of functional groups that help to improve the bioavailability, and BBB permeability. Besides, proper structure activity relationship (SAR), bioactivity guided isolation of the natural product and experiments from complete *in vitro/vivo* models to drug discovery, is the prime necessity of this era, in order to get rid of the unwanted side effects of synthetic drugs that are readily available. Through SAR, the relationship between the chemical structure of compounds and its biological activity can be assessed, and easily determine the particular chemical group inducing a particular biological effect. In Chinese dragon’s blood chalcone, homoisoflavanone and flavone were found to suppress the neuroinflammatory process in neurodegenerative diseases [130]. By SAR analysis, it was observed that the active moiety (1-(2,4-dimethoxy-phenyl)-propenone), substituent position in the phenyl ring was the key component in imparting the anti-inflammatory effect of pyrazole chalcones [131]. SAR can be very helpful in confirming the lead potential compounds for future drug discovery. 

## 6. Plant Extract or Phytochemical for Neurological *C*omplications

Several medicinal plants are well-reported to have efficacy against a variety of neurological disorders; however, very few have reached clinical trials. Curcumin, a well-studied medicinal spice, is reported to have anti-inflammatory and anti-neurodegenerative effects in *in vitro* and *in vivo* experimental models, and also in patients with dementia (AD pathologies in particular) [132,133,134,135]. Similarly, resveratrol, a naturally occurring phenolic compound widely present in grapes and berries, such as blueberries, mulberries and raspberries, is reported to have protective effects against several neurological complications in cellular and physiological systems, and in clinical trials [136,137,138]. Ginger (*Zingiber officinale*) and its constituents, 6-gingerol, 6-shogaol, 6-paradol, zingerone, *Ginkgo biloba* extract, quercetin, berberine, and apigenin are effective for AD in cell cultures and in animal models [139,140,141,142,143,144,145]. Spicatoside A, which is one of the active compounds of *Liriope platyphylla,* also possess anti-inflammatory and memory repairment in animal models of dementia [146]. *Scutellaria baicalensis* Georgi and *Lindera neesiana* extract have potential anti-inflammation and neuroprotective properties, and can be used during AD progression [120,147,148]. Broccoli extract enriched in sulforaphane attenuates neuroinflammation, and because of its neuroprotective role, it can be used for combating neurodegenerative diseases, such as AD, PD, MS, and ischemia [149]. Fatty acids isolated from stem bark of *Sorbus commixta* have been found to have neuroprotective activity [150]. *Bacopa monnieri* attenuated symptoms of anxiety and epileptic disorders in animal models [142]. The neuroprotective efficacy of these phytochemicals clearly indicate that they could be strong candidates for the management of neurological complications.

Neurological disorders have a very complex pathology, in which a huge number of genes or proteins are involved. Inflammation is the major cause of such conditions, and induction of TNF-*α* secretion and its secondary toxicity leads to chronic neuroinflammation. In TNF-*α*-mediated neuroinflammation, several secondary targets are also involved. Preliminary screening of the extracts from the dietary substances or edible natural products controlling those sub-targets of TNF-*α* can help design the multi-targeted phytochemical or extract formulation, which could be an alternative strategy for the downregulation of neuro-inflammatory cascades. Dietary products with traditional uses against neuroinflammation could provide greater safety and affordability to patients. From the preliminary screening of the dietary extracts for their anti-inflammatory effects, preparation of optimal mixture of extracts can help design the functional food as well. Determination of these effects could help for *in vivo* and clinical studies. Utilization of such medicinal foods not only promote the use of nutraceuticals medically, either to prevent or to ameliorate the negative effect of inflammation, but also help to financially discover a new drug that could have an appealing strategy for neurological complications.

## 7. Conclusions

TNF-*α* is a key cytokine involved in many neurological disorders, ranging from simple inflammation to dementia, depression, and infectious disorders. For decades, inhibitors or neutralizing antibodies for TNF-*α* have been an important alternative for the treatment of such conditions. However, their effect is still not entirely satisfactory, and therefore, screening for natural phytochemicals that can control TNF-*α* activity is essential for the treatment of various types of inflammatory disorders in human organs, especially in the brain. 

As many of the commercially available anti-inflammatory drugs, including TNF-*α* inhibitor, have severe side effects, the use of natural products are considered to be the most desired candidates for drug discovery against various biological ailments. From the studies conducted so far, candidate phytochemicals could inhibit TNF-*α* signaling and activity through TNFR1 or could block TACE and thereby inhibit inflammation, apoptosis, degeneration, cancer, and immune disorders in the brain. In addition, phytochemicals that activate TNF-*α*/TNFR2 activity could improve conditions by inhibiting inflammation and related disorders. This review summarizes phytochemicals against TNF-*α* and their neuroprotective properties in various neurodegenerative diseases. Even though the use of phytochemicals as anti-inflammatory agents has been widely studied in *in vitro* and *in vivo* studies, clinical trials using these natural phytochemicals are still limited due to their complex structures and solubility issues. Future studies can focus on overcoming this solubility issue by designing better formulation through nanoparticle formulation, drug micelle formation, prodrug formulation, and designing semisynthetic derivatives with the addition of functional groups that help improve bioavailability and BBB permeability. Hence, developing natural phytochemical drugs against TNF-α may be a potential therapeutic intervention in neurodegenerative diseases. Conclusively, we strongly suggest that neuroinflammation relating to TNF-α and TNFR1-mediated signal transduction may be potential therapeutic targets against various neurodegenerative and age-related diseases. TNF-*α* inhibitors from natural products might ameliorate neuroinflammation and cognitive dysfunction in neurodegenerative disease patients. Therefore, phytochemicals may ameliorate TNF-*α*-induced neurological impairments through anti-inflammatory effects, and it could be an effective dietary supplement and nutraceuticals against brain aging and neurodegenerative diseases. In addition, these investigations may provide a novel therapeutic candidate in the treatment and prevention of AD and other neuroinflammatory diseases. In the present review, we summarized the evidence supporting the beneficial role of anti-TNF-*α* phytochemicals to prevent or slow the progression of various neurodegenerative diseases to modulate TNF-*α* induced neuroinflammation.

## Figures and Tables

**Table 1:** Commercially available TNF-α inhibitors prescribed for several inflammatory disorders.

TNF-*α* Inhibitor	Disease(s)	Toxicity/Side Effect	Mechanism of Action	Reference(s)
Adalimumab	CD, retinitis pigmentosa, psoriatic arthritis	Increased risk of infections, lymphoma	Blocks the effects of TNF-*α*, reduces oxidative stress	[62,63]
Apratastat	RA	Less effective, dropped from clinical trial	TACE and MMP inhibitor	[64]
Certolizumab	RA, CD	Meningococcal meningoencephalitis, palmoplantar pustulosis	Inhibits soluble TNF-*α* binding	[52,53]
Golimumab	RA, ankylosing spondylitis, psoriatic arthritis	Bacterial and viral infection, fungal infection, tuberculosis	Prevents TNF-*α* binding with TNFR1 and TNFR2	[54]
Infliximab	CD, psoriasis, cognitive improvements, AD	Parkinsonism	Binds with high affinity to soluble and transmembrane forms of TNF-*α*, progressive MS	[54,55]
Minocycline	ALS, MS, AD, stroke, TBI, spinal cord injury	Dizziness, vertigo, lightheadedness	TNF-*α* synthesis inhibition	[56,65]
Thalidomide	Multiple myeloma, CD, Behcet’s disease, HIV, lupus, leprosy	Congenital abnormalities, birth defects, sensorimotor peripheral neuropathy, somnolence, rash, fatigue, constipation	Increases TNF-*α* degradation	[66,67]
GW3333	RA, other inflammation	NA	TACE and MMP inhibitor	[64]
BMS-561392	RA, other inflammation	NA	Specific TACE inhibitor	[64]
Etanercept	Acute and chronic stroke, post-stroke cognitive impairment, chronic brain injury	NA	Inhibits natural TNF-*α*, TNF blockade	[68,69]

**Abbreviations:** CD, Chorn’s disease; RA, rheumatoid arthritis; AD, Alzheimer’s disease ALS, amytrophic lateral sclerosis; MS, multiple sclerosis; NA, not applicable.

**Table 2:** TNF-*α* secretion-inhibiting phytochemicals that play beneficial roles in controlling the pathogenesis of several neuronal disorders.

Medicinal Plant	Active Compound	Compound Full Name	Mechanism of Action	Disease/ Pathogenesis	Experiment Model	Reference(s)
*Wasabia japonica*	AITC (isothiocyanate)	3-isothiocyanatoprop-1-ene	Inhibits JNK/NF-κB signaling and inhibit TNF-*α* secretion	Neuroinflammation	*In vitro*	[77]
*Zingiber officinale*	6-Shogaol (phenols)	(E)-1-(4-hydroxy-3-methoxyphenyl)dec-4-en-3-one	Attenuates LPS-induced TNF-alpha secretion, protects dopaminergic neurons	Neuroinflammation, PD	*In vitro, In vivo*	[78,79]
*Glycine max*	Genistein (isoflavone)	5,7-dihydroxy-3-(4-hydroxyphenyl)chromen-4-one	Inhibits ERK activation and NF-κB regulation by blocking the cleavage of IκB*-*α	Inflammation, muscular dystrophy	*In vitro*	[80,81,82]
*Brassica oleracea*	Quercetin (aglycon)	2-(3,4-dihydroxyphenyl)-3,5,7-trihydroxychromen-4-one	Inhibits nuclear translocation of NF-κB and phosphorylated Akt	MPTP-induced neurotoxicity	*In vitro, in vivo*, human subject	[83,84]
*Allium fistulosum*	Kaempferol (flavonoid)	3,5,7-trihydroxy-2-(4-hydroxyphenyl)chromen-4-one	Inhibits TLR4 and corresponding downstream activation of NF-κB, JNK, p38 MAPK, and Akt	Neuroinflammation	*In vitro*	[83]
*Psidium guajava*	Apigenin (flavone)	5,7-dihydroxy-2-(4-hydroxyphenyl)chromen-4-one	Attenuates the upregulation of NF-κB gene	PD	*In vitro, In vivo*	[85]
*Citrus paradisi*	Naringenin (flavanone)	**(2S)-5,7-dihydroxy-2-(4-hydroxyphenyl)-2,3-dihydrochromen-4-one**	**Inhibits iNOS/NO, decreases α-synuclein expression and neuroinflammation in PD**	Neuroinflammatory injury	*In vitro*	[86]
*Brassica oleracea*	Myricetin (flavonoid)	3,5,7-trihydroxy-2-(3,4,5-trihydroxyphenyl)chromen-4-one	Attenuates the activation of the MAPK and NF-κB signaling pathways	AD, PD	*In vitro*	[87]
*Rhus verniciflua*	Butein (polyphenol)	(E)-1-(2,4-dihydroxyphenyl)-3-(3,4-dihydroxyphenyl)prop-2-en-1-one	Inhibits the production of IL-1*β*, IL-6, and TNF-*α*	Neuroinflammation, neurotoxicity	*In vitro*	[88,89]
*Citrus sinensis*	Hesperetin (flavanone)	(2S)-5,7-dihydroxy-2-(3-hydroxy-4-methoxyphenyl)-2,3-dihydrochromen-4-one	Inhibits iNOS expression and TNF-*α* production	Neuroinflammatory injury	*In vitro*	[90,91]
*Scutellaria baicalensis*	Wogoni n(flavone)	5,7-dihydroxy-8-methoxy-2-phenylchromen-4-one	Alteration of JAK/STAT pathways	AD, PD	*In vitro*	[92]
*Maclura pomifera*	Morin (flavonol)	2-(2,4-dihydroxyphenyl)-3,5,7-trihydroxychromen-4-one	Inhibits NF-κB- and AP-1-mediated transcription and phosphorylation of MAPKs and Akt	Neuroinflammation, AD	*In vivo*	[93,94]
Honey, propolis	Chrysin (hydroxyflavone)	5,7-dihydroxy-2-phenylchromen-4-one	Inhibits iNOS, COX2, NO	Neuroinflammation	*In vitro*	[95]
*Zanthoxylum armatum*	Eudesmin (lignan)	3,6-bis(3,4-dimethoxyphenyl)-1,3,3a,4,6,6a-hexahydrofuro[3,4-c]furan	Suppression of NF-κB	Inflammation	*In vitro*	[96]
*Magnolia* *fargesii*	Magnolin (lignan)	(3S,3aR,6S,6aR)-3-(3,4-dimethoxyphenyl)-6-(3,4,5-trimethoxyphenyl)-1,3,3a,4,6,6a-hexahydrofuro[3,4-c]furan	Suppression of NF-κB, NO, PGE2	Inflammation	*In vitro*	[97,98]
*Magnolia* *officinalis*	Honokiol (lignan)	2-(4-hydroxy-3-prop-2-enylphenyl)-4-prop-2-enylphenol	Inhibits the phosphorylation of PI3K/Akt/MAP kinases, NF-κB, and CB2 receptor	Neurodegenerative diseases (e. g. AD)	*In vitro*	[99,100]
*Panax ginseng*	Ginsenoside Rg1 (triterpene glycosides)	(2R,3R,4S,5S,6R)-2-[[(3S,5R,6S,8R,9R,10R,12R,13R,14R,17S)-3,12-dihydroxy-4,4,8,10,14-pentamethyl-17-[(2S)-6-methyl-2-[(2S,3R,4S,5S,6R)-3,4,5-trihydroxy-6-(hydroxymethyl)oxan-2-yl]oxyhept-5-en-2-yl]-2,3,5,6,7,9,11,12,13,15,16,17-dodecahydro-1H-cyclopenta[a]phenanthren-6-yl]oxy]-6-(hydroxymethyl)oxane-3,4,5-triol	Reduces the levels of IL-1*β*, IL-6, and TNF-*α*	D-galactose-induced aging (related to AD)	*In vito, in vivo*	[101]
*Panax ginseng*	Ginsenoside Rb2 (triterpene glycoside)	(2S,3R,4S,5S,6R)-2-[(2R,3R,4S,5S,6R)-4,5-dihydroxy-6-(hydroxymethyl)-2-[[(3S,5R,8R,9R,10R,12R,13R,14R,17S)-12-hydroxy-4,4,8,10,14-pentamethyl-17-[(2S)-6-methyl-2-[(2S,3R,4S,5S,6R)-3,4,5-trihydroxy-6-[[(2S,3R,4S,5S)-3,4,5-trihydroxyoxan-2-yl]oxymethyl]oxan-2-yl]oxyhept-5-en-2-yl]-2,3,5,6,7,9,11,12,13,15,16,17-dodecahydro-1H-cyclopenta[a]phenanthren-3-yl]oxy]oxan-3-yl]oxy-6-(hydroxymethyl)oxane-3,4,5-triol	Suppresses TNF-*α* production via NF-κB inhibition	AD, PD, MS	*In vivo*	[102]
*Nicotiana tabacum*	Nicotine (alkaloid)	3-[(2S)-1-methylpyrrolidin-2-yl]pyridine	Immune modulation, alteration of MYD88/NF-κB downstream pathway	AD, PD, MS	*In vitro,* In vivo and in patients	[103,104,105,106]
*Berberis vulgaris*	Berberine (alkaloid)	16,17-dimethoxy-5,7-dioxa-13-azoniapentacyclo[11.8.0.02,10.04,8.015,20]henicosa-1(13),2,4(8),9,14,16,18,20-octaene	Downregulates acetylcholinesterase and inhibits the activation of the NF-κB signaling pathway	AD	*In vitro* and *In vivo*	[107,108,109]
*Capsicum annuum*	Capsaicin (alkaloid)	(E)-N-[(4-hydroxy-3-methoxyphenyl)methyl]-8-methylnon-6-enamide	Inhibits glial activation-mediated oxidative stress and neuroinflammation	PD	*In vitro* and *In vivo*	[110,111,112]
*Piper methysticum*	Kavalactones (polyketide)		Reduces intracellular oxidative stress	AD, stroke	*In vitro* and *In vivo*	[113,114,115]
*Vitis vinifera*	Resveratrol (Polyphenol)	5-[(E)-2-(4-hydroxyphenyl)ethenyl]benzene-1,3-diol	Upregulates the expression of the suppressor of SOCS-1	PD	*In vitro* and *In vivo*	[116,117,118,119]
*Lindera neesiana*	Koaburaside (ether)	(2S,3R,4S,5S,6R)-2-(4-hydroxy-3,5-dimethoxyphenoxy)-6-(hydroxymethyl)oxane-3,4,5-triol	Inhibits inflammatory mediators, pro-inflammatory cytokines in LPS-activated microglia, prevents neuronal death	AD, PD, MS lesions	*In vitro*	[120]
*Patrinia saniculaefolia*	Nardostachin (iridoid)	[(1S,4aS,6S,7R,7aS)-6-hydroxy-7-methyl-1-(3-methylbutanoyloxy)-1,4a,5,6,7,7a-hexahydrocyclopenta[c]pyran-4-yl]methyl 3-methylbutanoate	Reduces cytokines, COX-2, and PGE2	Inflammatory disorders such as neuroinflammation	*In vitro*	[121]
*Perilla frutescens*	Magnosalin, Andamanicin (neolignans)	1-[(1R,2R,3R,4R)-2,3-dimethyl-4-(2,4,5-trimethoxyphenyl)cyclobutyl]-2,4,5-trimethoxybenzene	Inhibits neuroinflammation and cell death	Degenerative disease	*In vitro*	[122]
***Petrosaspongia*** *nigra*	Petrosaspongiolid M	[(1S,3R,4aR,4bS,6aS,10aS,10bS,12aS)-3-(2-hydroxy-5-oxo-2H-furan-3-yl)-4b,7,7,10a-tetramethyl-1,3,4,4a,5,6,6a,8,9,10,10b,11,12,12a-tetradecahydronaphtho[2,1-f]isochromen-1-yl] acetate	Reduces PGE2, NO, and TNF-*α* levels	Acute and Chronic Inflammation	*In vivo*	[123]
*Salvia miltiorrhiza*	Tanshinone (diterpene)	1,6-dimethylnaphtho[1,2-g][1]benzofuran-10,11-dione	Selectively suppresses pro-inflammatory gene expression and partially decreased anti-inflammatory genes expression	Neuropathic pain, Neuroinflammation	*In vitro* and *in vivo*	[124,125]
Vitamin A	Retinoic acid (terpenes)	(2E,4E,6E,8E)-3,7-dimethyl-9-(2,6,6-trimethylcyclohexen-1-yl)nona-2,4,6,8-tetraenoic acid	Inhibits TNF-*α* and iNOS in (A*β*) or LPS-induced microglia-mediated neuroinflammation	AD, activated microglia-mediated brain disorders	*In vitro*	[126]
*Allium sativum*	Diallyl sulfide (sulfide)	3-prop-2-enylsulfanylprop-1-ene	Suppress pro-inflammatory cytokines by decreased ROS production through-induced PI3K/Akt and reduced NF-κB and AP-1	Inflammation, Allergy	*In vitro*	[74]
Curcuma longa	Curcumin (polyphenol)	(1E,6E)-1,7-bis(4-hydroxy-3-methoxyphenyl)hepta-1,6-diene-3,5-dione	Reduction of NF-κB mediated transcription	Inflammation,	*In vitro*, in vivo and in human	[70,75]
